# Digital variance angiography enables up to 80% reduction in stationary acquisition radiation dose during prostatic artery embolization: a prospective randomized trial

**DOI:** 10.1007/s00330-025-12235-3

**Published:** 2026-01-03

**Authors:** Leona S. Alizadeh, Thomas J. Vogl, Walid Rafi, Marta Caruso, Richárd Elek, Szabolcs Osvath, Andreea Nica, Leon D. Gruenewald, Ibrahim Yel, Aynur Goekduman, Vitali Koch, Mirela Dimitrova, Tommaso D’Angelo, Christian Booz

**Affiliations:** 1 Department of Diagnostic and Interventional Radiology, University Hospital Frankfurt, Frankfurt, Germany; 2 Department of Diagnostic and Interventional Radiology, Bundeswehr Central Hospital Koblenz, Koblenz, Germany; 3 Doctoral College of Theoretical and Translational Medicine, Semmelweis University, Budapest, Hungary; 4 Department of Biophysics and Radiation Biology, Semmelweis University, Budapest, Hungary; 5 Scientific Department, Kinepict Health Ltd., Budapest, Hungary; 6 Diagnostic and Interventional Radiology Unit, BIOMORF Department, University Ospital “Policlinico G. Martino”, Messina, Italy

**Keywords:** Radiation protection, Prostatic disease, Therapeutic embolization, Image quality enhancement, Digital image processing

## Abstract

**Objectives:**

Digital variance angiography (DVA) has demonstrated superior image quality compared to digital subtraction angiography (DSA), but its potential remains underexplored for complex procedures.

**Materials and methods:**

This prospective randomized controlled trial enrolled 70 patients (mean ± SD age: 69.23 ± 8.7, range 53–96) undergoing PAE between January and October 2023. Patients were randomized to normal dose (ND) DSA (*n* = 35) or ultra-low dose (ULD) DSA (*n* = 35), with the latter reducing target detector dose by 72% for stationary acquisitions only. Radiation dose analysis was limited to stationary acquisitions, as only these series were acquired using the modified low-dose protocol. Dose-area product (DAP), contrast-to-noise ratios (CNR), and visual image quality of DSA and DVA images were compared. Three experienced interventional radiologists conducted a randomized, blinded 5-point Likert evaluation of large and small vessels, tissue blush, and background noise. Statistical analysis included Mann-Whitney tests, Spearman correlation, Kendall Tau B, and Bangdiwala’s B for interrater agreement.

**Results:**

The ULD protocol reduced stationary acquisition-related DAP by up to 80% compared to controls (580 ± 66 vs 2872 ± 396 µGym²/patient, *p* < 0.001). DVA showed significantly higher CNR in both groups, with median CNR_DVA_/CNR_DSA_ ratios of 3.85 in ND and 4.60 in ULD. DVA images achieved significantly higher scores for small vessels and tissue blush visualization (*p* < 0.001; CNR: 4.10 vs 2.88; Likert rating: 2.79 vs 1.58).

**Conclusion:**

DVA enables substantial radiation dose reduction in PAE while maintaining superior image quality versus DSA, potentially improving angiographic safety and efficacy.

**Key Points:**

***Question*** *DVA is useful in reducing dose and enhancing image quality, yet it was not validated for complex angiographic procedures*.

***Findings*** *DVA provides better qualitative and quantitative image quality than DSA with reduced dose for prostatic artery embolizations*.

***Clinical relevance*** *DVA significantly improves image quality in Prostatic Artery Embolization compared to DSA, which allows up to 80% reduction in radiation burden to patients and interventionalists alike, while it also maintains diagnostic image quality*.

**Graphical Abstract:**

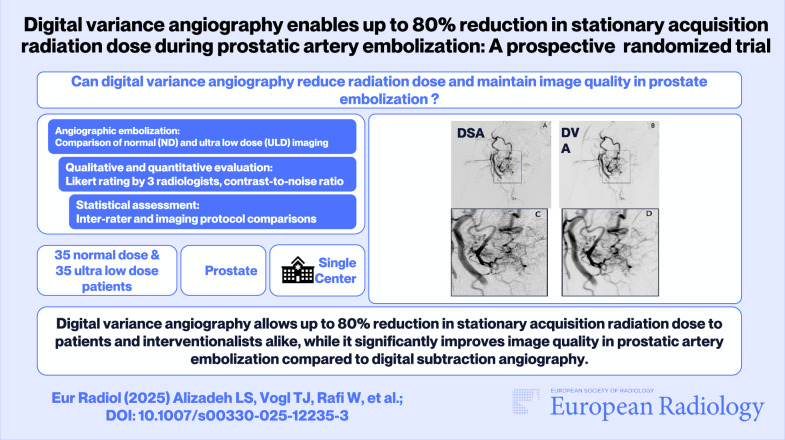

## Introduction

Interventional radiology (IR) procedures often require prolonged imaging for precise anatomical visualization and therapeutic endpoint monitoring, which can lead to substantial radiation exposure for both patients and staff [1–3]. According to the ALARA principle (as low as reasonably achievable), radiation dose and image quality must be carefully balanced for each procedure.

Prostatic artery embolization (PAE), used for the treatment of benign prostatic hyperplasia (BPH), represents one of the most technically demanding IR interventions and is associated with particularly high radiation doses [3–6]. PAE has gained prominence given that BPH is one of the most commonly treated conditions in older men [1, 7]. The procedure’s acceptance is underscored by endorsements from the British National Institute for Health and Care Excellence guidelines since 2018 and the American Urological Association’s guidelines for BPH-related lower urinary tract syndromes (LUTS) since 2023 [8, 9]. Despite advantages, such as good response and low complication rates, concerns remain regarding the significant radiation exposure inherent to PAE. In PAE, stationary angiographic acquisitions, such as digital subtraction angiography (DSA), account for more than 70% of the total procedural radiation dose and the use of significant amounts of iodinated contrast media (ICM), potentially increasing the risk of nephropathy and loss of renal function in vulnerable patients [5, 6, 10].

Digital variance angiography (DVA) is a novel angiography image reconstruction technique that has emerged as a promising alternative to conventional DSA in IR [11–18]. DVA calculates pixel intensity variance over time, providing enhanced vascular contrast and signal clarity while reducing background noise. Previous studies on the lower limbs [11–14, 17, 18], carotids [15], and liver [19] have demonstrated DVA’s superiority for these selected indications in image quality compared to DSA. PAE is considered to be a very complex angiographic procedure. In the context of PAE, analyses indicated that DVA provides a higher CNR and an image quality reserve, visualizing complex vascular anatomy and precisely targeting embolization endpoints [16]. Building on this evidence, our prospective randomized controlled trial aimed to investigate whether DVA’s documented quality reserve could be facilitated to significantly reduce radiation dose during PAE procedures.

## Materials and methods

### Ethical approval

The Institutional Review Board approved the study (ref. 2022-941), and all patients provided written informed consent.

### Patient inclusion and study design

**Fig. 1:**
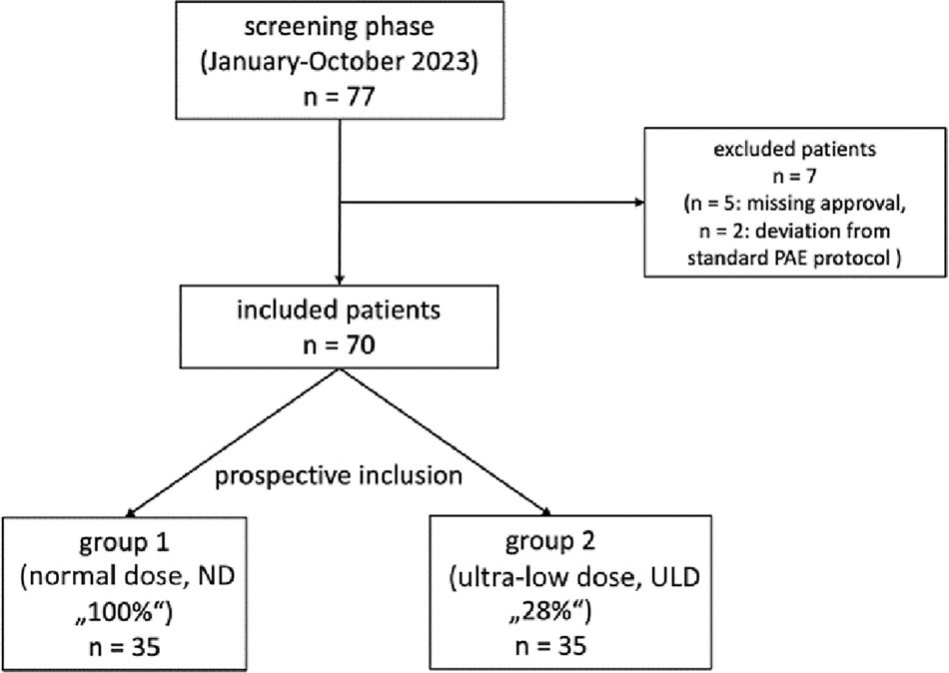
Flow chart of the study. Symptomatic patients with BPH, referred to our institute for PAE between January and October 2023, were consecutively screened for eligibility and prospectively included after randomization. PAE, Prostatic artery embolization; BPH, benign prostatic hyperplasia

In 2023 (January–October), 70 male patients were prospectively enrolled and randomized into two equal groups (*n* = 35; each) using block randomization with a fixed block size. Eligibility and exclusion criteria are detailed in Fig. 1. Patients underwent PAE. DVA images were generated from the same raw data as DSA, allowing direct paired comparison. These images were displayed simultaneously with DSA on a split-screen in the angiography suite. The first group received a standard radiation dose (normal dose, ND) and optimized image guidance during the PAE procedures. The second group underwent PAE procedures with reduced radiation (ultra-low dose, ULD). Sample size was determined based on an expected large effect size for radiation dose reduction (Cohen’s *d* ≥ 0.8), as shown by previous studies and preliminary data, assuming an alpha level of 0.05, a statistical power of 80%.

### PAE procedure

“PErFecTED”—PAE technique [20] was applied: a unilateral puncture of the right femoral artery was performed, followed by the placement of a 5 F introducer sheath. Initial angiography of the aortic bifurcation was performed using a 5 F pigtail catheter. Depending on the individual vascular anatomy, a crossover maneuver was performed, and the left internal iliac artery was visualized using either a 4 F Sidewinder-1 or a 4 F Cobra catheter. Superselective catheterization of the prostatic artery (PA) was then achieved using a 2.4 F Progreat microcatheter (Terumo, Tokyo, Japan). The same steps were repeated on the right side, primarily employing the Sidewinder-1 catheter for right internal iliac artery selection. To minimize the risk of non-target embolization through vulnerable collaterals to the rectum and genitals, the PA was reached superselectively with the 2.4 F microcatheter. Embolization was performed as distally as possible, aiming for complete stasis. Bilateral embolization was achieved in all procedures using 100–300 µm embolic spheres (Embosphere® Microspheres; Merit Medical). All procedures were performed on an outpatient basis, and no severe complications were observed. All interventions were executed by the same interventional radiologist (T.J.V.) with 21 years of experience.

### Image acquisition

PAE was performed using a state-of-the-art angiography suite (ARTIS pheno®, Siemens Healthineers). In the ND group, standard pre-installed DSA acquisition protocols (“CARE aorta” and “CARE pelvis”) were used, with automatic exposure control active for dose optimization. The mean tube voltage was 84 kV, the mean tube current 321 mA, and copper filtration 0.298 mm Cu. In the ULD group, the same protocols were applied with a reduced target detector dose of 28%, resulting in a mean tube voltage of 76 kV, a mean tube current of 242 mA, and copper filtration increased to 0.709 mm Cu. The system’s automatic exposure regulation (CARE Dose) adjusted the tube current and filtration in real time according to patient attenuation to maintain consistent image brightness. Procedural aspects were unchanged; only stationary acquisitions (SAs) were optimized in the ULD group. An automated injector (Medrad Mark 7 Arterion; Bayer AG) was used to administer 15–30 mL of ICM (Ultravist® 370; Bayer) at a flow rate of 3–10 mL/s per injection. The specific injection rate was adjusted according to vessel caliber and catheter position. The same contrast injection protocols and volumes were applied in both the ND and ULD groups to ensure comparability. All images were retrieved as unprocessed and uncompressed raw data and reconstructed for both the ND and ULD groups. Mask images were manually selected by an interventional radiologist (21 years of experience). The DVA images were reconstructed using the Kinepict Medical Imaging Tool, version 4.0 (Kinepict Health Ltd.), installed on a dedicated local workstation connected to the angiography suite via the institutional PACS. Unprocessed, uncompressed DICOM datasets from the angiography system were automatically transferred to the workstation immediately after acquisition. DVA reconstruction was performed offline but in near real-time (processing time < 10 s per series), using the same raw data as the corresponding DSA series. The DVA and DSA images were then displayed side by side on the angiography console (split-screen), allowing immediate visual comparison and evaluation during the procedure. This workflow did not require modification of the vendor’s acquisition protocol or hardware integration.

### Radiation dose

DAP values and procedural data were obtained from the dose report files. Because the ULD protocol applied only to SAs, fluoroscopy and road map acquisitions were not modified as part of this protocol and thus excluded from our study. The total SA-related DAP was calculated for each patient, and these cumulative values were used for group comparisons, along with the number of series, the total number of pulses, the average dose per pulse, and the total exposure time.

### Quantitative image quality

Regions of interest (ROI) were defined on vessels and the background using ImageJ (v.2.0.0-rc-68/1.52e, Creative Common License, NIH). The vascular and background ROIs were placed in pairs. Contrast-to-noise ratio (CNR) values were calculated for all ROI pairs individually [12]. CNR_DVA_/CNR_DSA_ ratios (*R*) for each corresponding DVA and DSA ROIs were calculated.

### Qualitative image quality

Three interventional radiologists (experience: 6, 8, and 10 years) performed a randomized and blinded rating of 70 DSA and DVA image pairs, generated from the same raw data, resulting in 210 individual comparisons between DSA and DVA series for each protocol. To avoid bias related to the varying number of stationary angiographic acquisitions among patients, one representative series per case was randomly selected for DSA–DVA comparison using the RAND() function and series numbers in Microsoft Excel. Regarding image quality assessment, “large vessels” were defined as the internal iliac artery and its first-order branches (diameters ≥ 2.5 mm), whereas “small vessels” included the PA and smaller IIA branches with diameters < 2.5 mm. Visibility of large and small vessels, tissue blush, and the level of background noise were assessed using a five-point Likert scale for diagnostic image quality: (1) non-diagnostic; (2) poor, with significant limitations; (3) average, allowing for adequate interpretation; (4) good; and (5) excellent visualization with optimal clarity. Additionally, image noise and artifacts were evaluated on a similar five-point Likert scale: (1) severe; disabling image interpretation (2) noticeable; moderately affecting the accuracy of image interpretation, (3) moderate; visible but not substantially impacting image reading, (4) minimal; allowing for good image interpretation, (5) no visible; perfect for image reading. An additional option was available if the structure was not present (for some cases of tissue blush). Only those images were included that were recognized and scored by all three raters.

### Statistical analysis

In this study, we compared DVA and DSA image quality ratings by subtracting the scores assigned to the 70 DVA-DSA image pairs. Interrater agreement was assessed using Bangdiwala’s B statistic, which provides a robust measure of agreement that is less affected by prevalence and category imbalances compared to the paradoxical behavior of Cohen’s kappa [21, 22]. To evaluate the statistical significance of image quality differences, we applied the Mann–Whitney *U*-test due to non-normal distribution. Fisher’s exact test was applied for patient distribution to check the equivalence of the groups. Consistency in ratings across raters was analyzed using Spearman’s correlation test, while interrater agreement was further quantified with Kendall’s Tau B coefficient. Statistical analysis was performed with Python 3.11, JASP 0.18, and Microsoft Excel.

## Results

A total of 70 male patients underwent PAE (mean age ± SD: 69.23 ± 8.7 years, range: 53–96 years), half of them with ND, the other half with ULD DSA protocol. The Fisher’s exact test confirmed no significant differences between the groups regarding demography (p = 0.84). Table 1 summarizes the detailed results of the demographic analysis. None of the patients received previous urological surgery of the prostate. Ninety-one (*n* = 64) of patients received alpha-1 inhibitors (Prazosin, Tamsulosin) prior to the PAE treatment, but they were classified as therapy refractory or showed progressive LUTS under medication.

**Table 1:** Demographic data table

	Group a	Group b
Group name suffix number	ND (100% dose, “ND”) (*n* = 35)	ULD (28% dose, “ULD”) (*n* = 35)
Age	71.41 (±9.1; 55–96)	67.23 (±8.3; 53–85)
Height [m]	1.78 (±0.07; 1.68–1.95)	1.78 (±0.07; 1.60–1.96)
Weight [kg]	82.25 (±13.11; 56–120)	81.11 (±16.36; 50–130)
BMI	25.77 (±3.28; 19.6–32.8)	25.52 (±4.01; 18.3–37)
Patient abdominal a.p. diameter [cm]	21.7 (±1.9; 16.87–25.87)	21.15 (±3.05; 16.9–29.94)
Prostate volume [mL]	154.13 (±66.3)	164.89 (±47.95)

*n* number of patients. Body mass index (BMI). Values are mean ± standard deviation, and range. Patient abdominal diameter was reported to mitigate bias from increased abdominal fat mass affecting radiation dose in the region of interest. *ND* normal dose, *ULD* ultra-low dose

### Radiation dose

**Fig. 2:**
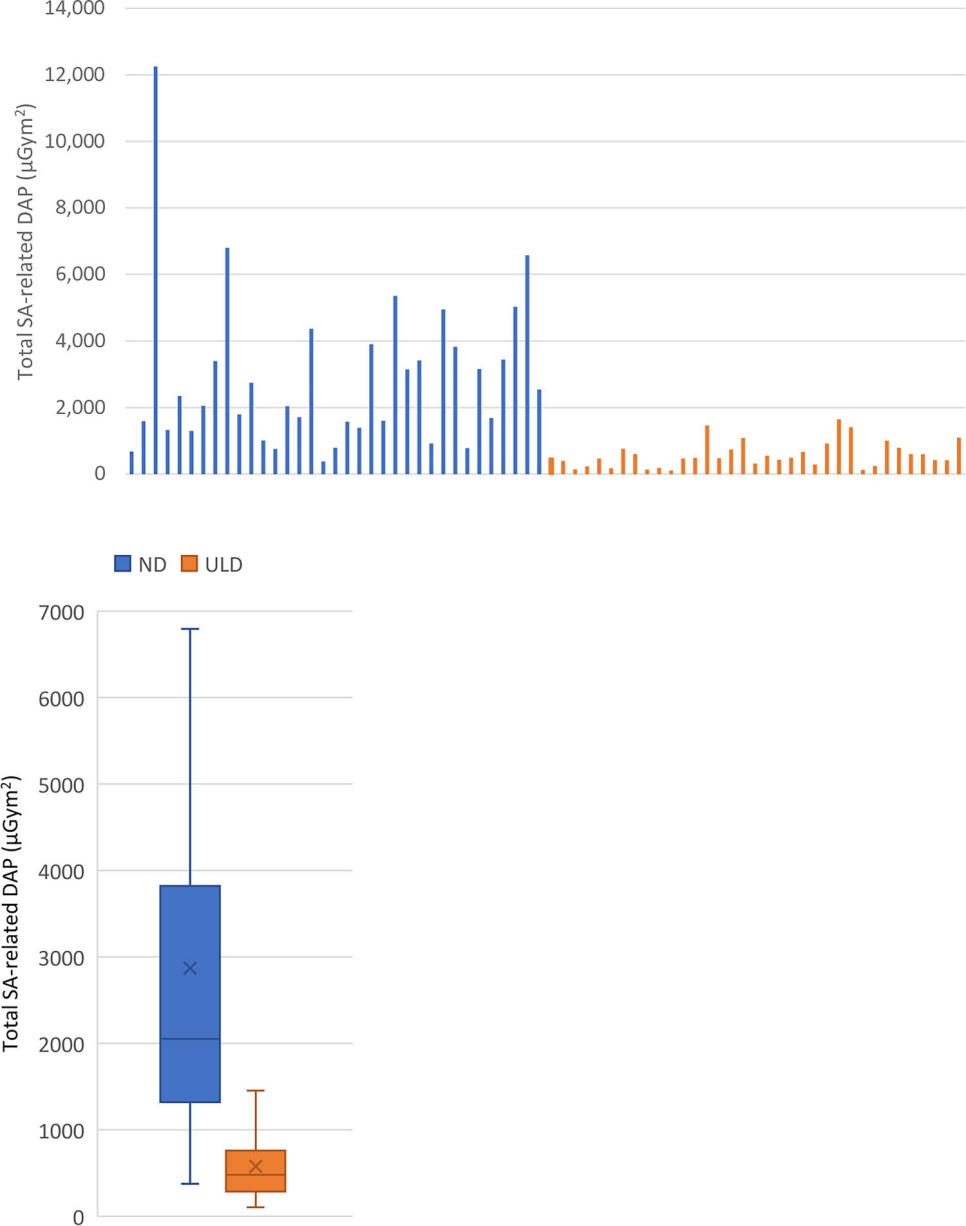
Cumulative SA-related DAP values. **A** Patients’ individual data. **B** The box and whisker plots show the mean (*x*), median (line), IQR (box), and bounding values (whiskers) of cumulative SA-related values for the ND (100%) and ULD (28%) groups. Outliers are not shown separately. DAP, dose-area product; SA, stationary acquisition; ND, normal dose; ULD, ultra-low dose

The mean SA-related cumulative DAP was 2872 ± 396 µGym^2^ in the ND group and 580 ± 66 µGym^2^ in the ULD group (Mann–Whitney test *p* < 0.001), which represents an 80% reduction in DAP. Similar reduction was observed in the median and interquartile range (IQR) values (ND group: 2052 (3631) µGym^2^; ULD group: 479 (747) µGym^2^, reduction by about 78%) (Fig. 2). There was no difference in the DAP values of other fluoroscopy-related radiation events. Figure 2 also shows the individual cumulative SA-related DAP values for each patient. Comparing the ND with ULD acquisitions hardware settings, as a result of decreasing the aimed detector dose in the acquisition protocols, the average tube voltage (kVp) was reduced from 84 to 76 kVp, while the Cu filtration became larger from 0.298 mm to 0.709 mm. Average tube current also decreased from 321 mA to 242 mA. Procedural differences for the ND and ULD groups are insignificant, as the number of series (*p* = 0.334), the total number of pulses (*p* = 0.601), and the average of total exposure times (*p* = 0.089) were not significantly different based on the Mann–Whitney *U*-test for the ULD and ND groups. The Mann–Whitney *U*-test showed no significant difference between groups in oblique or cranio-caudal C-arm angulations (*p* = 0.304). However, the total DAP (*p* = 0.001) and the average dose per pulse (*p* = 0.001) for these groups are significantly different, based on the same test.

### Quantitative image quality

For the CNR evaluation, 896 ROI pairs were evaluated. DVA provided significantly higher CNR values in each group (Table 2); the median (IQR) R value was 3.85 (1.87) in the ND group and 4.60 (1.81) in the ULD group (Wilcoxon signed rank *p* < 0.001). The ULD protocol had a stronger influence on the CNR of DSA images, as the median CNR decreased by 32% whereas the reduction was only 25% for the DVA images.

**Table 2:** Results of the quantitative image quality comparison

Group	Image type	CNR		WSR p	R		OSW p
		Median	IQR		Median	IQR	
ND (“100%”)	DSA	5.60	4.49	< 0.001	3.85	1.87	< 0.001
	DVA	22.75	17.92				
ULD (“28%”)	DSA	3.83	3.13	< 0.001	4.60	1.81	< 0.001
	DVA	17.00	14.26				

The CNRs were calculated using the same ROI sets on the corresponding DSA and DVA images. CNR values were compared using the Wilcoxon signed rank test (WSR), the significance of R ratios (CNR_DVA_/CNR_DSA_) was determined by the one-sample Wilcoxon test (OSW), and the significance was set to *p* < 0.05 in all cases.

*DSA* digital subtraction angiography, *DVA* digital variance angiography, *IQR* interquartile range, *ND* normal dose, *ROI* region of interest, *ULD* ultra-low dose

### Qualitative image quality

For interrater agreement, Bangdiwala’s B values evaluation is summarized in Table 3. The Mann–Whitney test showed that raters A, B, and C agreed that ND-DVA and ULD-DVA provided better visualization of large and small vessels, as well as tissue blush, compared to DSA (*p* < 0.001). However, for background noise, the results varied (*p* = 0.047 for rater A, 0.002 for B, and 0.085 for C).

**Table 3:** Bangdiwala’s B values representing the pairwise interrater agreement among the three raters, calculated for each evaluation criterion of images recorded using the ND (top) and ULD (bottom) protocols

	Large vessels	Small vessels	Tissue blush	Noise
ND protocol				
Rater A vs Rater B	0.548 (substantial)	0.734 (almost perfect)	0.476 (substantial)	0.309 (moderate)
Rater A vs Rater C	0.244 (moderate)	0.199 (moderate)	0.243 (moderate)	0.181 (moderate)
Rater B vs Rater C	0.384 (substantial)	0.401 (substantial)	0.480 (substantial)	0.318 (moderate)
ULD protocol				
Rater A vs Rater B	0.427 (substantial)	0.558 (substantial)	0.492 (substantial)	0.523 (substantial)
Rater A vs Rater C	0.290 (moderate)	0.361 (substantial)	0.342 (moderate)	0.425 (substantial)
Rater B vs Rater C	0.345 (moderate)	0.528 (substantial)	0.527 (substantial)	0.626 (substantial)

*ND* normal dose, *ULD* ultra-low dose

The Spearman test showed a strong correlation in all pairwise comparisons of raters’ evaluations of large and small vessels and tissue blush (*p* < 0.001) for both ND and ULD acquisitions. However, no correlation was found for background noise ratings between raters A and B (*p* = 0.643) or A and C (*p* = 0.166) in ND images, while a moderate correlation was observed between raters B and C (*p* = 0.033). For ULD images, background noise ratings showed strong correlations across all rater comparisons (*p* < 0.001 for A–B, *p* = 0.015 for A–C, and *p* = 0.015 for B–C). The Kendall Tau B coefficient for pairwise comparisons of interrater agreement was chosen over other measures due to its suitability for ordinal data and its smaller sensitivity to imbalances in prevalence and marginal distributions. For both ND and ULD ratings, raters showed strong agreement on large and small vessels and tissue blush (*p* < 0.001). For background noise in the ND-DSA dataset, disagreement was observed (*p* = 0.640 for rater A–B, 0.161 for A–C, and 0.036 for B–C). In contrast, ULD-DVA ratings showed strong agreement between raters A and B (*p* < 0.001), with slight disagreement between A–C and B–C (both *p* = 0.016).

**Fig. 3:**
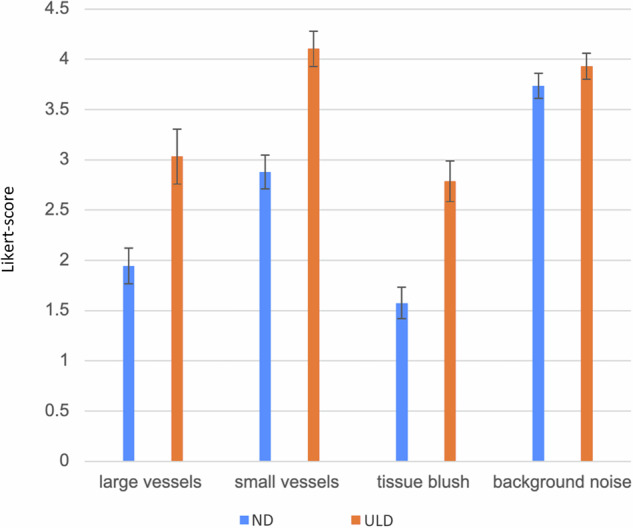
Bar plot of qualitative image quality score results for each criterion: the visibility of large vessels, small vessels, tissue blush, and the level of background noise on a 5-point Likert scale. DVA, digital variance angiography; DSA, digital subtraction angiography; ND, normal dose; ULD, ultra-low dose

**Fig. 4:**
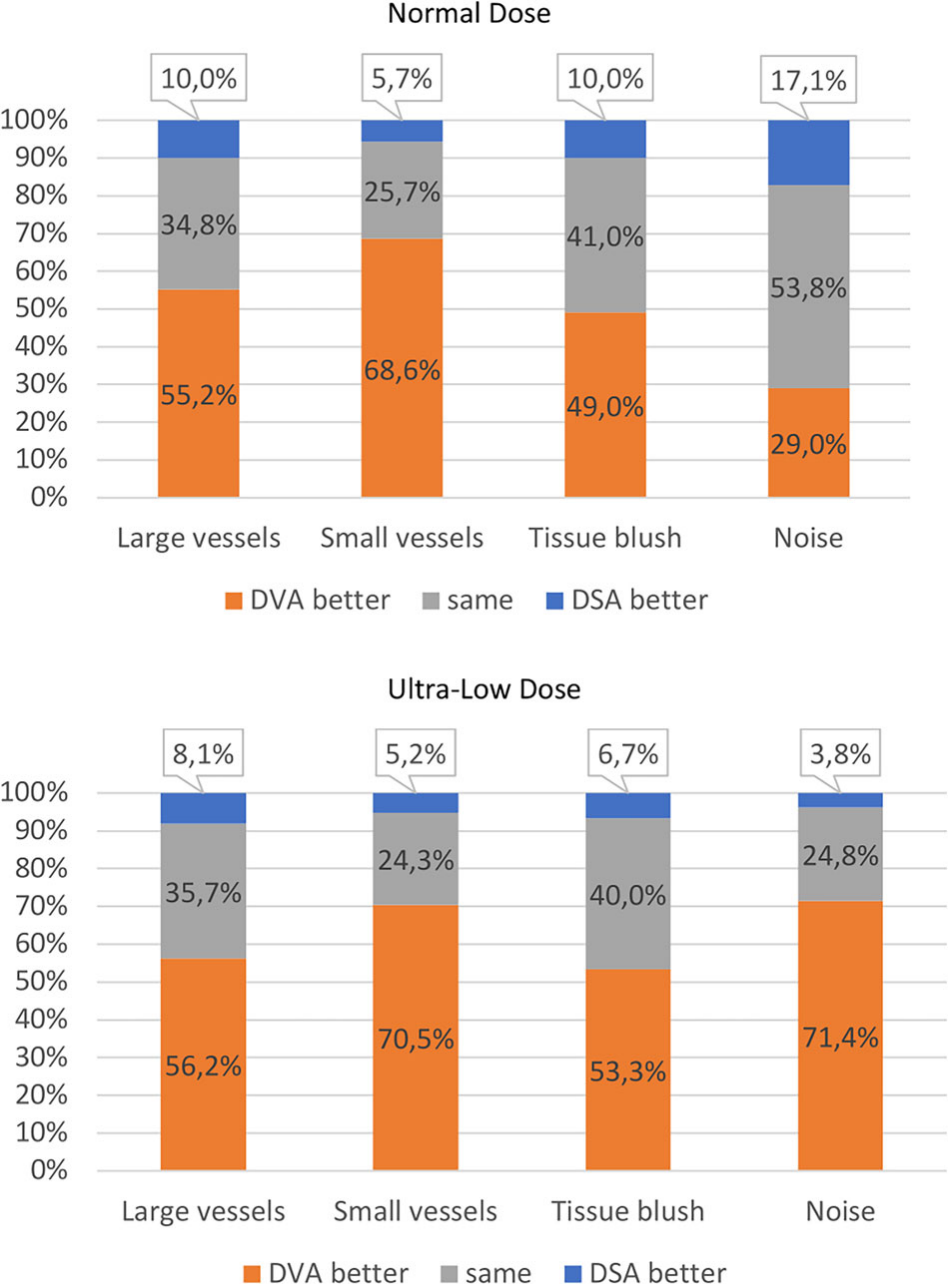
These stacked column charts illustrate the qualitative preferences of three raters for image quality across four evaluation categories: large vessels, small vessels, tissue blush, and background noise. The plot compares the qualitative preference of each rater between the imaging datasets from ND (**A**) and ULD (**B**) imaging protocols, DVA image processing (DVA, orange), and DSA (blue). Instances where no difference was observed are labeled as “same” with a gray color. DSA, digital subtraction angiography; DVA, digital variance angiography; ND, normal dose; ULD, ultra-low dose

For large vessels, the mean score for ND-DVA (2.94 ± 0.14) was significantly higher than ND-DSA (1.94 ± 0.088), while ULD-DVA (3.033 ± 0.136) also exceeded ULD-DSA (2.00 ± 0.10). Similar trends were observed for small vessels, where ND-DVA (3.98 ± 0.10) and ULD-DVA (4.11 ± 0.09) achieved notably higher scores compared to ND-DSA (2.88 ± 0.08) and ULD-DSA (3.03 ± 0.08) (*p* < 0.001). Tissue blush also demonstrated a marked difference, with ND-DVA (2.41 ± 0.12) and ULD-DVA (2.79 ± 0.10) scored significantly better than ND-DSA (1.58 ± 0.08) and ULD-DSA (1.84 ± 0.08) (*p* < 0.001). For background noise, while ND-DVA (3.83 ± 0.08) and ULD-DVA (3.93 ± 0.06) still outperformed ND-DSA (3.73 ± 0.06) and ULD-DSA (3.00 ± 0.07), the differences were less pronounced. Figure 3 visually represents the Likert-rating score distributions in all categories. Figure 4 illustrates a strong reader preference for DVA over DSA across all categories and dose levels when the scores for each series are directly compared. For ND-DVA, readers consistently rated it superior in large vessels (116 cases), small vessels (144 cases), tissue blush (103 cases), and noise (61 cases), compared to ND-DSA (21, 12, 21, and 36 cases, respectively). Similarly, ULD-DVA received higher ratings for large vessels (118 cases), small vessels (148 cases), tissue blush (112 cases), and noise (150 cases), significantly outperforming ULD-DSA (17, 11, 14, and 8 cases, respectively).

## Discussion

Our study aimed to determine whether the image quality advantage of DVA, as previously observed in a retrospective analysis of PAE interventions [16], could be effectively leveraged to reduce radiation exposure during this complex procedure in line with the ALARA principle [23]. PAE is an effective alternative to transurethral resection of the prostate in the treatment of BPH [3]. However, prolonged intervention times in PAE can result in substantial radiation exposure, posing a risk of both stochastic and deterministic tissue damage [6, 23–28]. Stochastic risk also extends to medical staff, especially in high-volume PAE centers [5, 29]. Previous studies reported 13,440 ± 6950 µGym² PAE procedural doses, of which 5820 ± 4830 µGym² were attributed to DSA [30]. More recent literature indicates even higher DAP values of 21,265 ± 19,358 µGym² from a cohort of 551 patients in interventions performed by an experienced interventional radiologist [31], while another study from 2020 reported a procedural DAP of 24,709 µGym² for six interventional radiologists with 5–15 years of experience following four years of training in PAE [26]. This prospective randomized controlled trial demonstrated that DVA can enable up to an 80% reduction in SA-related radiation dose for PAE while maintaining superior image quality compared to ND-DSA (*p* < 0.001). The reduction corresponds to an average of 79.5%, though it applies exclusively to SAs. Although the overall procedural dose was approximately 12% lower in the ULD group compared to the ND group (*p* < 0.001), this reduction does not accurately reflect the specific effect of DVA, as fluoroscopy and cone-beam CT parameters were unaltered and the total dose remains highly dependent on individual anatomical and technical variability.

**Fig. 5:**
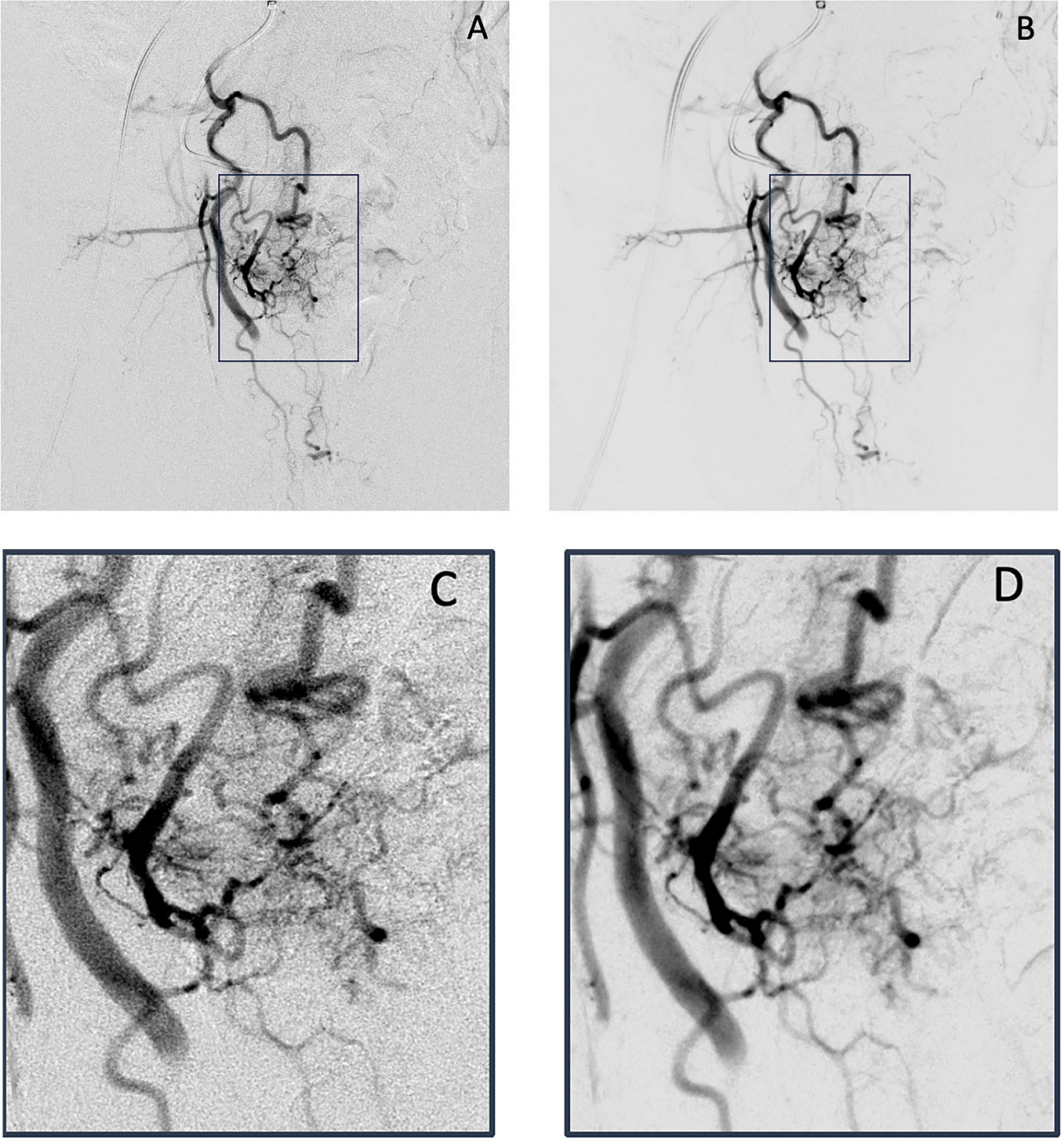
Images in a 72-year-old patient from the ND group, showing a super selective angiogram with super selective catheterization of the right PA using a 2.4 F micro catheter. The DSA and DVA images were rated with mean scores of 3.3 for DSA and 3.8 for DVA, for excellent depiction of small vessels and 2.4 vs. 3.7 tissue blush as visualized in the magnification images, **C**, **D** of the image area that was marked in **A**, **B** (boxes). In direct comparison, the DSA image shows a slightly higher level of visual image noise, masking the tissue blush in **C**, compared to a higher visibility in **D**. DSA, digital subtraction angiography; DVA, digital variance angiography; ND, normal dose; PA, Prostatic artery

**Fig. 6:**
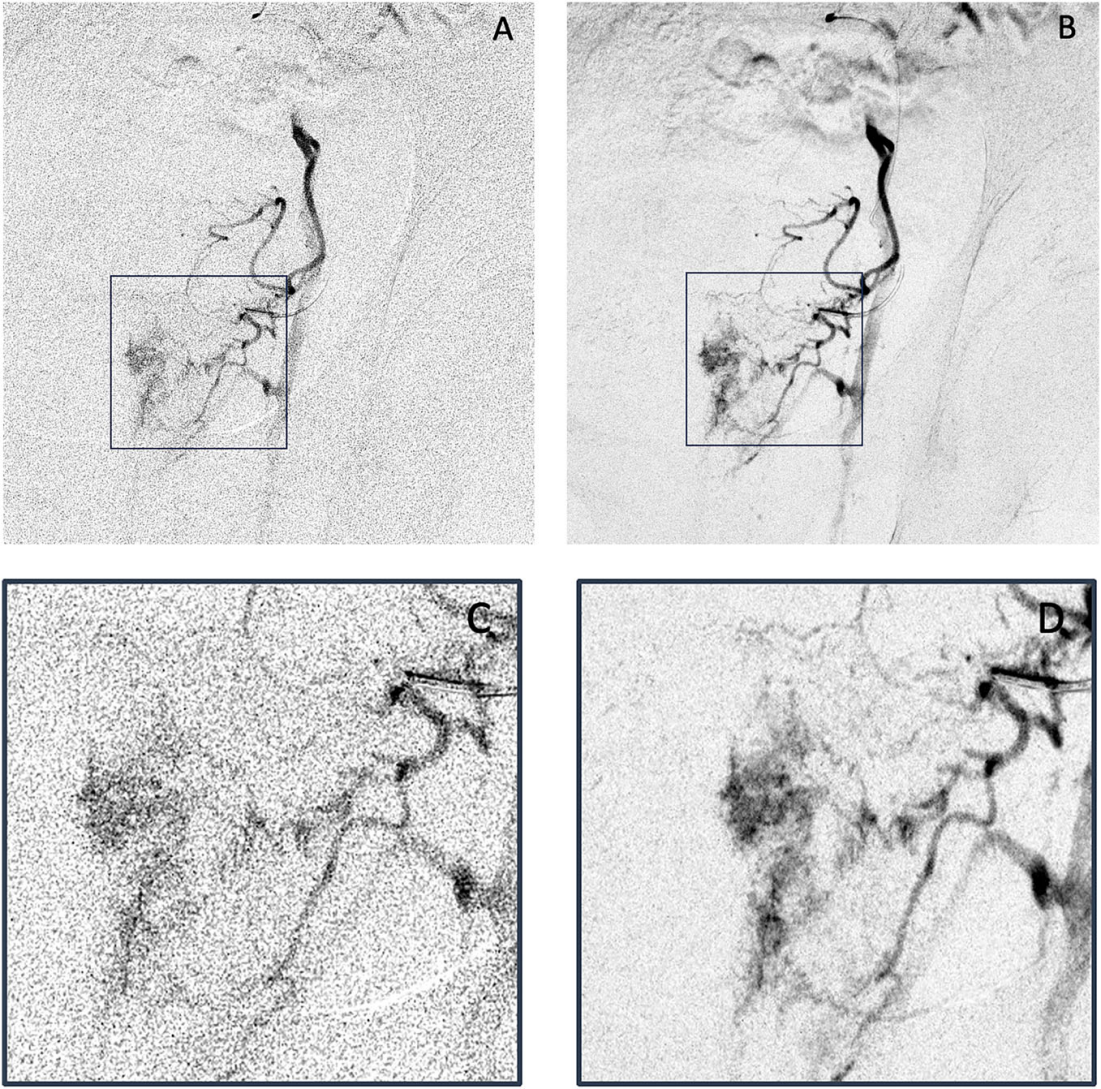
Representative example of reduced dose DSA (**A**) and DVA (**B**) images in a 63-year-old patient from the ULD group. The images show a super selective angiogram with super selective catheterization of the left PA using a 2.4 F micro catheter. Magnification of the region of interest (boxes in **A**, **B**). **C**, **D** Emphasize the increase in image noise when using the DSA reconstruction method on a ULD acquisition. The DSA image was rated with a mean score of 1.3 in the visual evaluation for background noise, due to the high level of visual image noise, and relatively low intravascular contrast. In direct comparison, the DVA image on the right scored with a mean of 3.4 points for background noise, due to a very good contrast and very low levels of visible image noise, exploiting the image quality reserve of DVA. DSA, digital subtraction angiography; DVA, digital variance angiography; PA, Prostatic artery; ULD, ultra-low dose

Quantitative and qualitative evaluations confirmed that DVA sustained superior image quality even with ULD protocols. In the ND group, DVA achieved approximately a four-fold increase in the CNR compared to DSA. For small vessels, DVA significantly outperformed DSA at both ND (mean score: 3.98 ± 0.10 vs 2.88 ± 0.08) and ULD (mean score: 4.11 ± 0.09 vs 3.03 ± 0.08) protocols (*p* < 0.001). Visual comparisons (#Figs. 5 and 6) clearly demonstrate the robustness of DVA; while DSA image quality deteriorated at ULD, DVA preserved diagnostic quality. Furthermore, improved scores for large vessels (ULD-DVA: 3.03 ± 0.14 vs ULD-DSA: 2.00 ± 0.10) prove enhanced vessel delineation and navigation in complex vascular anatomies. The most notable differences were found in small vessels and tissue blush, where DVA consistently achieved significantly higher scores, especially at the ULD settings. Background noise was notably lower in DVA images (ND-DVA: 3.83 ± 0.08; ULD-DVA: 3.93 ± 0.06) compared to DSA, where scores dropped significantly (from 3.73 ± 0.06 at ND to 3.00 ± 0.07 at ULD). Figures 5 and 6 further illustrate these findings, demonstrating the quality reserve of DVA in ULD relative to DSA. In this context, the term “image quality reserve” refers to the robustness of DVA in maintaining diagnostic image quality despite substantial reductions in radiation dose, reflecting its ability to preserve vascular detail under low-signal conditions. A lack of consensus is evident for the background noise among raters, apparently due to the already acceptable level of noise in any case.

These findings emphasize DVA’s capacity to enhance visualization of fine vascular structures and perfusion, which is critical for procedures like PAE, where effective embolization requires superselective catheterization with 2.3 F microcatheters [32–34]. The superior scores for DVA in large and small vessels suggest improved navigation in complex vascular anatomies associated with PAE [32, 33, 35].

Conclusively, the image quality reserve of DVA effectively reduces radiation exposures without compromising image quality. In the context of modern IR, visualization of the smallest arteries, dangerous collaterals, e.g., to the rectum and genital area, may avert severe damage by DVA, due to dislocation of embolic agents and consecutive tissue necrosis [3, 4, 7].

Importantly, while this study focused on PAE, a procedure noted for its technical complexity, its implications are broader. The ability of DVA to achieve substantial radiation dose reductions while preserving image quality positions it as a valuable tool for other angiographic procedures—such as those for benign tumors, peripheral vascular disease, or transarterial embolization for myomas or periarticular pain [36]. Moreover, previous studies showed DVA can reduce contrast agent by 50% in carotid angiography [15] and allowed 60–70% reduction of DSA-related radiation dose in lower limb angiography [11, 18]. This combination of lower radiation and/or contrast requirements suggests potential benefits for both younger and at-risk patients.

Our study has limitations that need to be addressed. Despite comparable patient characteristics (e.g., BMI, age, abdominal anterior-posterior diameter), variability in prostatic vascular anatomy introduces procedural heterogeneity that may affect radiation doses. To mitigate this, our analysis focused solely on SAs rather than the full procedural dose, which includes fluoroscopy. The overall procedural radiation dose was only slightly reduced (≈12%) because the ULD protocol modified only SAs, while fluoroscopy and roadmap settings all remained unchanged. Given the strong influence of anatomical variability, procedural complexity, and fluoroscopy duration on total exposure during PAE, our analysis focused on SAs to isolate the protocol’s direct effect.

Although DVA reconstruction is performed on an external vendor-independent workstation with only a brief processing delay of 2–10 s, deeper software integration could further streamline workflow, improve user handling at the operating table, and potentially enable advanced features such as real-time roadmap use or fluoroscopy overlay in the future.

Given the high radiation exposure associated with complex angiographic procedures, our findings suggest that DVA could be widely applicable to other interventions, such as tumor embolization, peripheral vascular disease treatments, and neurointerventional procedures. The ability to maintain image quality at ultra-low radiation doses could improve patient safety and reduce operator exposure across multiple specialties.

## Conclusion

DVA enables up to 80% radiation dose reduction for SAs in complex interventional procedures such as PAE while maintaining high image quality. Our study demonstrates that with the reduction in radiation dose, image quality is preserved, supporting DVA’s role as a safer alternative to DSA in PAE and other angiographic procedures. Future research should explore its applications in additionally reducing contrast agent use and further validating its benefits in real-world clinical settings and implications for vulnerable patient cohorts.

## Abbreviations

ALARA: As low as reasonably achievable
BMI: Body mass index
BPH: Benign prostatic hyperplasia
CNR: Contrast-to-noise ratio
DAP: Dose-area product
DSA: Digital subtraction angiography
DVA: Digital variance angiography
ICM: Iodinated contrast media
IQR: Interquartile range
IR: Interventional radiology
LUTS: Lower urinary tract symptoms
ND: Normal dose (imaging protocol or group)
PA: Prostatic artery
PAE: Prostatic artery embolization
ROI: Region of interest
SA: Stationary acquisition
ULD: Ultra-low dose (imaging protocol or group)
